# Predicting perceived quality of life through social trust, physical activity, and sense of happiness in Iran: Moderating role of gender

**DOI:** 10.1002/hsr2.2121

**Published:** 2024-05-20

**Authors:** Rajabi Gilan, Jamal Mohamadi, Shirin Zardoshtian, Neda Sarabi, Naseri Palangard, Mehdi Khezeli

**Affiliations:** ^1^ Sociology Department, Faculty of Humanities and Social Sciences University of Kurdistan Sanandaj Iran; ^2^ Social Development & Health Promotion Research Center, Health Institute Kermanshah University of Medical Sciences Kermanshah Iran; ^3^ Department of Sport Management, Faculty of Sport Science Razi University Kermanshah Iran; ^4^ Department of Social Sciences Education Shahid Modarres Campus, Farhangian University ilam Iran

**Keywords:** gender, happiness, physical activity, social capital, quality of life

## Abstract

**Background and Aims:**

In Iran, few studies have addressed the moderating effect of gender on determinants of quality of life. This study aimed to determine the effect of social trust, physical activity, and sense of happiness on the perceived quality of life, considering the moderating role of gender.

**Methods:**

This was a population‐based cross‐sectional study. The statistical population were men and women aged 16 years and above in five western provinces of Iran. The final sample size was estimated to be 1268 people calculated through the correlation coefficient estimation formula, and finally 1185 questionnaires received. Data were analyzed using SPSS and AMOS software.

**Results:**

The mean score of quality of life among women was significantly higher than that of men (*p* < 0.001). The direct standardized coefficient showed that the sense of happiness (*β* = 0.40), social trust (*β* = 0.20), and physical activity (*β* = 0.12) had a significant effect on the perceived quality of life (*p* < 0.001). Also, gender had a moderating role in the relationship between sense of happiness and perceived quality of life (*z*‐score = 3.246, *p* < 0.001). Totally, 21% of the changes in perceived quality of life were explained by three main variables. The main variables were stronger explanatory factors in men (*R* = 0.26) than in women (*R* = 0.17).

**Conclusions:**

The final model showed that sense of happiness had the most direct effect on the perceived quality of life, moderated by gender. Considering that the quality of life among men was lower than that among women and the direct effect of happiness on quality of life was more among men than that among women, it is suggested that gender‐based health promotion interventions with an emphasis on men be carried out to strengthen the sources of happiness to improve quality of life.

## 1 INTRODUCTION

Following social development and improvement of people's well‐being, attention to the concept of quality of life (QoL) has grown significantly.^1^, ^2^ During the past decades, improving the QoL has been one of the main goals of development policies at the national and international levels.^3^ QoL is a multilevel concept, and perceived QoL refers to how people perceive and evaluate different aspects of their lives, such as marriage, interpersonal relationships, work, leisure activities, and health.^4^

To improve the interventions that promote the QoL, it is necessary first to know its predictors, determinants and related factors.^5^ Previous studies have suggested that personal and social factors affect the QoL, such as mental health, physical health, lifestyle, gender, marriage, nutrition, interpersonal relationships, job, and education.^6^ However, it is not possible to investigate the QoL of people unless we should consider social issues.^7^ Despite the costs that are spent to improve the QoL, the importance of many main social factors such as social capital (SC) has been neglected.^8^

SC is one of the most appealing concepts of social sciences used in the field of public health, which emphasizes on two main elements, firstly, trust between social agents and second, participation in social organizations.^9^ Social trust (ST) is a cognitive aspect of SC according to which people shape their relationship with others on moral values such as commitment, honesty, and responsibility. The previous studies showed that SC and trust can have significant effects on people's QoL.^10^, ^11^

Another variable related to QoL is exercise and physical activity (PA). PA provides people with different bodily benefits, such as improving organ function, reducing the risk of diseases, improving body composition, and weight management.^12^ Research have shown that sports and PA are not only effective on mental health, physical, and social adaptation in different periods of life, but also improve the socialization process of people. These studies have also emphasized on the role of PA in increasing happiness, self‐confidence, and mental health, and in reducing depression and anxiety.^13^

Sens of happiness (SH) is one of the components of QoL, and it has been introduced by the World Health Organization as part of the concept of health. Research show that SH as a multidimensional concept is influenced by individual and cultural factors; it has also a strong influence on many dimensions of human life and is a main facilitator in the development of societies.^14^ Many theorists define SH as mental health and use the terms interchangeably. SH refers to people's cognitive and emotional satisfaction with their life, expressing positive emotions and the absence of negative sentiments.^15^

Gender is a key factor in the subjective well‐being of citizens. The results of a study showed the existence of gender gaps in most European Union countries; it also showed the positive gaps which indicate the existence of higher levels of mental well‐being for men.^16^ Another study in Asia also showed that the health status and social service needs are highly dependent on gender.^17^ In Iran, many studies have examined the role of gender related to QoL or its predictor. There is a paradox in some studies, men report higher QoL than women despite lower life expectancy and more health risks. In the study by Rezaei et al. in Kermanshah using the EuroQoL Visual Analogue Scale (EQ‐VAS), women reported more QoL than men.^18^ In another study in Kermanshah, Karyani et al. found that women had lower levels of health‐related quality of life (HRQoL) and female gender was a significant predictor of lower QoL in society.^19^ Another study in southern Iran showed that women had weaker HRQoL than men in all aspects of SF‐36 except the emotional role, and after adjusting for other sociodemographic factors, female gender independently reduced both mental and physical aspects of HRQoL.^20^ The results of another study on adults in Shiraz, Iran showed that the HRQoL of women was significantly lower than that of men.^21^ In contrast, Ghasemi et al. found no significant difference in the HRQoL of men and women in Kermanshah, except for the physical pain subscale.^22^ Ghafari et al. also reported no gender differences in the QoL of men and women, except for the subscale of physical performance.^23^

Social inequalities and its objective example, gender inequality, are influenced by the structures of the political systems and cultures. In sociological terms, gender inequality means unequal social distribution of wealth, power, and benefits between men and women.^24^ The consequences of gender inequality at micro, medium, and macro levels justify the necessity of research in this field. There are many cases that show fundamental changes in the beliefs of people and government officials, and its effects have been manifested in social norms and the new gender identity in Iran. Iranian women in recent decades have experienced new values and identities and have gained more advanced education. This is why, in recent years, women in Iran oppose gender stereotypes that assign inferior positions to women and cause inequalities and limitations in their daily lives.^25^ Iranian women, especially in the last decade, are moving gender boundaries and taboos and changing collective SC in favor of their gender.^26^ In recent years, after continuous efforts, women have received the right to enter football stadiums to watch live matches.^27^ Also, women are trying for gender equality for the benefit of the quality of their sexual life.^28^

Nevertheless, some gender stereotypes still exist both in the minds of the people and in Iranian social institutions For example, Sadeghi et al. suggested that gender discrimination is primarily caused by the division of labor and gender roles in two public (male) and private (female) spheres of social life.^29^ Thus, gender is one of the obvious forms of social distinctions that lead to heterogeneity and inequality in social life. In this regard, the evidence in Iran shows that stereotypes and social inequalities, for example, in sports and physical activities still exist for women. Afrouzeh et al. showed that there are unequal educational and training opportunities, coverage restrictions, psychological insecurity, and unequal media coverage to the detriment of women in Iran.^30^

Obtaining information about the QoL of different groups of society can be a reliable foundation for evaluating health and QoL interventions.^4^ Considering the gender discrimination and inequalities mentioned above, the lack of studies on the effect of gender on the QoL,^31^ and the severe economic and social problems caused by sanctions and poor structures in Iran,^32^ It seems that it is necessary to conduct a study on the QoL and its determinants in Iran.

Also, considering that the west of Iran as the setting of this study is one of the deprived areas in terms of development indicators,^33^ and probably the QoL of people in these areas is different from other areas of Iran, so this study was conducted in five western provinces in Iran with two main objectives: first, to investigate the effect of ST, PA, and SH on QoL, and second, to investigate the moderating role of gender in the relationship between the main research variables.

### 1.1 Research hypothesis

1. ST, PA, and SH have a significant effect on perceived QoL.
2. Gender has a moderating effect on the relationships between ST, PA, and SH with perceived QoL.

## 2 METHODS

### 2.1 Study design

This is a population‐based cross‐sectional study. The statistical population of this research was urban women and men aged 16 years and older in western Iran (living in the cities of Kermanshah, Sanandaj, Ilam, Hamedan, and Khorramabad with a population of about 2.5 million people).^34^ The sample size was calculated with the following formula:

$$C\left(r\right)=\frac{1}{2}\ln\left(\frac{1+r}{1-r}\right),$$

$$n=\frac{\left(Z_{{1-}_{2}^{\alpha }}+Z_{{1-}_{2}^{\beta }}\right)}{C\;\left(r^{2}\right)}+3.$$

In which, (*r*) was the correlation coefficient between the main variables in previous studies.^8^, ^35^, ^36^, ^37^ By replacing the correlation coefficient in the formula *C*(*r*) was obtained and then the sample size (*n*) calculated for each variable. The final sample size was estimated to be 1268 people, taking into account the design effect of 1.2. The quota of each city was calculated according to the population of the cities (Kermanshah 483, Hamedan 283, Sanandaj 215, Khorramabad 187, and Ilam 100 samples). A multistage sampling method was used, based on which municipal districts were listed in each city. Then, two neighborhoods were randomly selected from each urban district. Then, according to the quota of each city in the total sample size, questionnaires were distributed in different areas of the neighborhood, including local shops, large shopping centers, and parks. Samples were recruited according to the convenience method. Before presenting the questionnaire, the research objectives were explained to the respondents. The respondents were assured about the confidentiality of the information, therefore, considering the limitations of self‐reporting in survey research, the respondents were asked to answer the research questions honestly.

The inclusion criteria were having consent to participate in the study, not having a physical disability and acute mental illness, and being over 16 years old. Finally, 1185 valid questionnaires were received and analyzed, indicating the response rate of 93.5%. At the time of questionnaire administration, the study objectives were explained, and the interviewer clarified any queries from the respondents. The questionnaire comprised of straightforward content with a limited number of questions, and the estimated time for completion was around 10 min. These factors collectively contributed to a higher response rate.

### 2.2 Measurements

The data collection tool in this study was a five‐part questionnaire in Persian language. The first part consisted of demographic information including age, sex, marital status (married, single, widowed), and education, which was classified in three levels: (1) subdiploma including elementary and high school education, (2) diploma degree, and (3) University education. The second part included three questions related to ST. These questions are a part of the SC questionnaire by Rafiey et al. which examines three aspects of ST: (1) trust in fellow tribes and religions, (2) trust in friends, colleagues, neighbors, and other residents of the neighborhood, and (3) trust in family members and close relatives.^38^ Each question is developed and scored on a five‐point Likert scale (1 = completely disagree to 5 = completely agree). The highest and lowest scores in this three‐question scale are 15 and 5, respectively. Rafiey and colleagues have assessed and confirmed the validity and reliability of this tool.^38^

Also in the present study Cronbach's *α* coefficient of the questionnaire was 0.825, which is an acceptable value.

To measure the level of participation in PA, we asked a question: “How much do you participate in sports and physical activities the last 4 weeks?” Responses were given as never, 1–3 times a month, 1–2 times a week, and almost every day, with total score from 1 to 4.^39^ We have clearly explained to the respondents that the meaning of sports and PA in this question is doing sports (including walking, climbing, cycling, aerobics, football, volleyball, etc.) and PA to maintain and promote health throughout the life cycle. Before the question, we provided the respondents with background explanations in the questionnaire. The fourth part of the questionnaire was a question about SH. Participants were asked which of the following is your true feeling the last 4 weeks. The answers were provided as 1: I feel extremely happy, 2: I am very happy, 3: I am a little happy, and 4: I am not happy at all. The validity and reliability of this scale has been confirmed in studies conducted in Iran.^40^ Then the questions recoded in the software and a higher score was determined as a sign of greater happiness. The final part of the questionnaire was the first question of the World Health Organization's QoL scale (WHOQoL‐BREF‐26), which is a general question about personal assessment of QoL the last 4 weeks. The answers are scored as (1) very bad, (2) bad, (3) neither good nor bad, (4) good, and (5) very good, where a higher score indicates a better QoL.^41^ Other studies used this single question and have confirmed its usability in measuring the QoL.^42^, ^43^, ^44^

### 2.3 Ethical statement

This study received ethical approval from the Ethics Committee of Kermanshah University of Medical Sciences (IR.KUMS.REC.1398.118). A written informed consent form was provided in the first page of printed questionnaires and was obtained from all participants.

### 2.4 Data collection and statistical analysis

Data were collected from October 15 to November 11, 2019 by trained and informed interviewers about the environment and social context of each district. The questionnaires were completed in public spaces and there were no challenges or obstacles to complete them in terms of gender. Statistical Package for the Social Sciences (SPSS‐18) (mean and standard deviation for quantitative description of variables and Pearson correlation test to assessing the correlations between main variables, and controlling the confounding variables through regression) and AMOS software (structural equation modeling (SEM), direct and indirect coefficients and Fisher's *z* test) were used for data analysis. Using SEM, goodness of fit and also the significance of variables effects were investigated. For this purpose, we used indices such as Chi‐square Mean/Degree of Freedom (CMIN/DF), Root Mean Square Error of Approximation (RMSEA), Comparative Fit Index (CFI), Goodness of Fit Index (GFI), and Adjusted Goodness of Fit Index (AGFI). Also, the direct, indirect, and total effects of variables and their significance were assessed. In this study, the dependent variable was QoL, and the independent variables were PA, SH, and ST. Considering the moderating role of gender, we implemented three SEM models in this research, including a model for women, a model for men, and a total model (Model fit in total model: NNFI = 0.94, CFI = 0.92, TLI = 0.94, RFI = 0.92, NFI = 0.95, AGFI = 0.91, GFI = 0.95, RMSEA = 0.039, CMIN = 24.97, DF = 9 CMIN/DF = 2.77) To ensure the validity of the measurement model, first‐order confirmatory factor analysis was used. In this method, all the measurement error coefficients were calculated and the direct and indirect path coefficients between the latent variables in the structural model were measured. The confidence level is 95%.

## 3 RESULTS

The total participants in this study were 1185 people, of which 54.3% were men and the rest were women. The mean and standard deviation (SD) of age of the respondents was 33.00 ± 12.69. In this study, 45.7% of respondents were married, 50.5% single, and 3.7% widowed or divorced. The findings on education showed that 51.4% of the participants had university education (Table 1).

**Table 1:** Demographic characteristics of the participant based on the main research variables (*N* = 1185).

Variable	Category	*N* (%)	Physical activity mean (SD)	Social trust mean (SD)	Sense of happiness mean (SD)	Quality of life mean (SD)
Age	16–24 years old	358 (30.1)	2.68 (0.96)	9.19 (3.42)	2.17 (0.83)	3.22 (1.03)
	25–34 years old	392 (33.7)	2.36 (1.04)	9.74 (2.76)	2.02 (0.81)	3.14 (0.98)
	35–44 years old	220 (18.5)	2.28 (1.06)	9.97 (2.86)	1.94 (0.73)	3.17 (0.95)
	45–54 years old	118 (9.9)	2.40 (1.11)	10.05 (2.86)	2.05 (0.77)	3.11 (0.93)
	55–64 years old	69 (5.7)	2.51 (1.12)	9.10 (3.27)	2.01 (0.68)	3.00 (1.15)
	65–75 years old	26 (2.1)	2.62 (1.16)	9.00 (3.66)	2.03 (0.66)	2.97 (0.91)
*p* Value			0.001	0.011	0.036	0.493
Sex	Male	643 (54.3)	2.61 (1.02)	9.70 (3.24)	1.99 (0.77)	2.61 (1.00)
	Female	542 (45.7)	2.28 (1.05)	9.43 (2.90)	2.14 (0.81)	3.25 (0.99)
*p* Value			0.001	0.150	0.001	0.003
Marital status	Single	599 (50.5)	2.57 (1.05)	9.55 (3.15)	2.05 (0.77)	3.16 (1.01)
	Married	542 (45.7)	2.38 (1.03)	9.73 (2.99)	2.09 (0.68)	3.19 (0.98)
	Widow	44 (3.8)	2.09 (1.07)	8.08 (3.03)	1.79 (0.66)	2.69 (0.98)
*p* Value			0.001	0.003	0.052	0.006
Literacy	Under diploma	188 (15.9)	2.28 (1.20)	9.16 (3.24)	1.96 (0.76)	2.95 (0.99)
	Diploma	388 (32.7)	2.38 (1.01)	9.41 (3.52)	2.99 (0.77)	3.06 (1.02)
	University	609 (51.4)	2.57 (1.01)	9.81 (3.52)	2.13 (0.81)	3.27 (0.97)
*p* Value			0.001	0.017	0.007	0.001
Total		1185 (100)	2.06 (0.79)	9.58 (3.09)	2.46 (1.05)	3.15 (1.00)

The descriptive results of the analysis on SH showed that 5.3% of the respondents expressed that they feel extremely happy and 19.1% are very happy, 52.1% are very little happy and 23.5% are not happy at all. Also, the descriptive results showed that the perceived QoL in 8% of people was very bad, 13.5% bad, 39.8% neither good nor bad, 32.7% good, and 6.1% very good. The results of the descriptive analysis on PA showed that 23.3% of people were inactive and did not have regular PA, while 19.5% exercised continuously and regularly. Also, 26.6% had PA 1–3 times a month, and 30.6% 1–2 times a week.

The Pearson correlation test showed that SH (*r* = 0.449), ST (*r* = 0.291) and PA (*r* = 0.214) have the highest correlation with QoL, respectively (Table 2).

**Table 2:** Matrix correlations among the main research variables (*N* = 1185).

Variables	Quality of life	Social trust	Physical activity	Sens of happiness
Quality of life	1			
Social trust	0.291**	1		
Physical activity	0.214**	0.146**	1	
Sens of happiness	0.449**	0.202**	0.176**	1

** Significant at 0.01 level.

**Figure 1:**
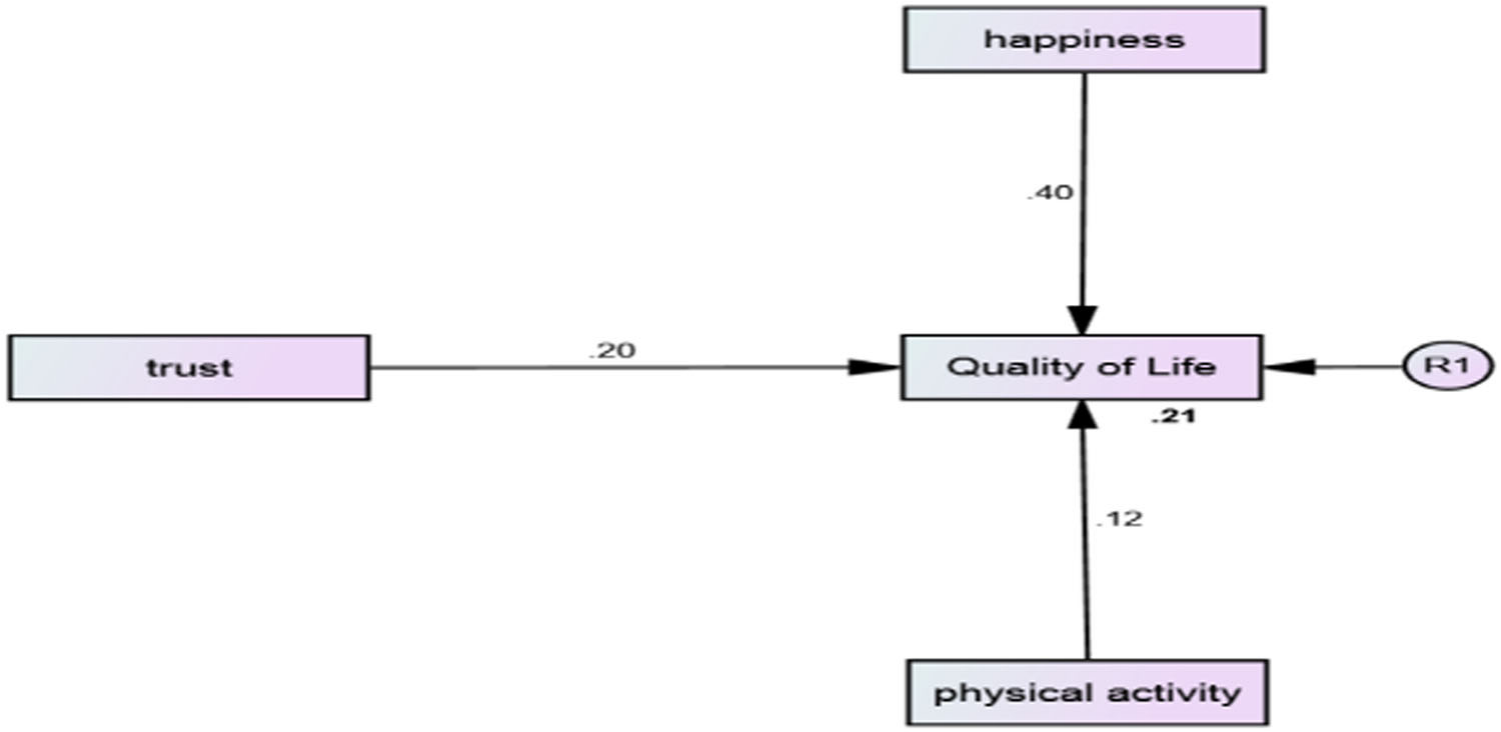
The Structural Equations Model (SEM) in the total sample.

The results showed that the direct standardized coefficients of the paths of SH (0.399, *p* < 0.001), PA (0.120, *p* < 0.001) and ST (0.201, *p* < 0.001) on perceived QoL were significant in the overall model. The *R* ^2^ index in the overall model, in men, and in women was 0.21, 0.26, and 0.17, respectively, indicating more explanatory power of variables in men than women and the whole sample (Table 3) (Figure 1). According to the total model fit summary, the Absolute Fit Indices of research model included the Goodness of Fit Index (GFI) = 0.95, and Adjusted Goodness‐Fit Index (AGFI) = 0.91; these appear at acceptable level. The Parsimonious Fit Indices included the Chi‐square Statistics (CMIN/DF) ratio = 2.77, and the Root Mean Square Error of Approximation (RMSEA) = 0. 039, which are also acceptable. This holds true also for the Comparative Fit Index (CFI) = 0.92. Tucker–Lewis index was (TLI) = 0.94, which was acceptable. It also should be noted that the direction and strength of these relationships were still significant after controlling possibly confounding variables including age, marital status, and education.

**Table 3:** Direct coefficients of the main variables on the quality of life (*N* = 1185).

Model	Paths	Direct coefficients		Error	*p* Value	*R* ^2^
		Nonstandard coefficients *B*	Standard coefficients *β*			
Total model	Happiness ➨ Quality Of Life	0.488	0.399	0.032	0.001	0.21
	Physical activity ➨ Quality Of Life	0.112	0.120	0.024	0.001	
	Social trust ➨ Quality Of Life	0.190	0.201	0.024	0.001	
Men	Happiness ➨ Quality Of Life	0.570	0.454	0.043	0.001	0.26
	Physical activity ➨ Quality Of Life	0.109	0.115	0.032	0.001	
	Social trust ➨ Quality Of Life	0.179	0.198	0.031	0.001	
Women	Happiness ➨ Quality Of Life	0.366	0.308	0.046	0.001	0.17
	Physical activity ➨ Quality Of Life	0.140	0.153	0.036	0.001	
	Social trust ➨ Quality Of Life	0.224	0.225	0.039	0.001	
Total model fit results		NNFI = 0.94, CFI = 0.92, RFI = 0.92, NFI = 0.95, AGFI = 0.91, TLI = 0.94 GFI = 0.95, RMSEA = 0.039, CMIN = 24.97, DF = 9, CMIN/DF = 2.77				

The findings showed that gender had a moderating effect on the path between SH and perceived QoL, while the effect of ST and PA on perceived QoL was not significant according to gender. In other words, gender does not moderate the effect of ST and PA on perceived QoL (*Z*‐score = 0.905, 0.649) (Table 4).

**Table 4:** The results of the moderating effect of gender on the relationship between happiness, social trust, and physical activity on quality of life (*n* = 1185).

Paths	Men		Women		*Z*‐ score
	Nonstandard coefficients *β*	*p* Value	Nonstandard coefficients *β*	*p* Value	
Happiness ➨ Quality Of Life	0.570	0.001	0.366	0.0001	3.246***
Physical activity ➨ Quality Of Life	0.109	0.001	0.140	0.0001	0.649
Social trust ➨ Quality Of Life	0.179	0.001	0.224	0.0001	0.905

*** Significant at 0.001 level.

## 4 DISCUSSION

This study was conducted to investigate the effect of ST, PA, and SH on perceived QoL considering the moderating role of gender, in western Iran. The results of the study showed that the SH had the most direct effect on the perceived QoL. A study in Colombia showed that happiness is a significant component of QoL that can lead to active aging ^45^, ^46^, ^47^, ^48^ The result of another study in the Spanish community showed that people with the highest level of QoL were more likely to claim to be happy.^49^ A global study by examining the indicators of the world's countries showed that the most and moderately happiest countries had a better QoL compared to the countries that had the lowest happiness index.^49^ Hosseiniamin et al. in Iran also showed that social happiness has a greater impact on QoL than social support.^49^ Studies in America have shown that happiness is more important than wealth in creating a desirable and satisfying life.^49^ SH as a positive emotion can facilitate interpersonal relationships and have wide positive consequences on cognition, social activity level, work activities, and health. Also, happiness empowers people to solve problems with creativity and innovation.^50^

Determining the effect of ST on QoL was another goal of this study, and the results showed a positive effect of PA on QoL. Rajabi Gilan et al. in Iran showed the relationship between the trust (relational dimension of SC) and QoL.^8^ Adedeji showed the significant direct effect of SC on QoL in a study among sub‐Saharan African immigrants living in Germany.^51^ Adedeji in another study also showed that SC is a strong predictor of QoL among immigrants.^52^

SC, as described by Putnam with the components of ST, reciprocity norms, and density of social networks has been identified in many studies as a factor related to the improvement of QoL.^53^ Studies have shown that SC provides various types of social support, including financial, emotional, informational, and practical support, among which emotional support clearly contributes to health and improving QoL.^54^ Giddens believes that emotional support is a protective shell that all people take refuge in when facing daily life problems.^55^ The development of group activities in the form of voluntary associations in society has facilitated participation, mutual trust, and the expansion of individual relationship networks and created communication opportunities. It seems that these social interactions strengthen emotional and social support and subsequently reduce daily tensions, increase life satisfaction, and improve QoL.^8^

This study investigated the effect of PA on QoL, indicating that PA had a weak, but direct and significant effect on the perceived QoL. Shafiei Alawijeh in a sample of Iranian elderly showed a similar finding.^13^ A systematic review of studies conducted worldwide concluded that for adults aged 18–65 years, PA strongly improves HRQoL compared with minimal or no treatment controls.^13^ Some studies from different parts of the world have also shown that there is a significant relationship between doing PA and life expectancy.^10^, ^56^, ^57^, ^58^ Considering the target group, studies in Iran have emphasized the relationship or effect of PA with/on QoL and health‐related QoL in the elderly,^59^ women,^60^ people with hypertension,^61^ and workers.^62^ Rafighi et al., showed that exercise had positive effects on self‐efficacy and as a result improved mental and physical health status and finally increased life satisfaction in women.^63^ Regular PA can improve people's QoL through the control of mental pressure. Both cases of very low and very high psychological pressure due to helplessness can reduce the QoL.^64^ PA can be used as one of the methods of managing mental pressure, both in raising and relieving mental stress.^65^ Tomporowski et al. in United States showed that regular PA can improve health and QoL by reducing depression, feelings of mental pressure and confusion, and increasing positive mood, energy, cognitive performance, and self‐esteem.^66^ The results of the present also showed that the direct effect of PA on the QoL was greater in women than in men. Considering the limitations in the field of women's sports in the study setting, it seems that the improvement of sports infrastructure for women can be effective in improving their QoL.

One of the hypotheses of this study was that gender has a moderating effect on the relationships between ST, PA, and SH with QoL. The results showed that gender had a moderating role only in the relationship between happiness and perceived QoL. To the best of our knowledge, this study is one of the few studies at the regional level in Iran that investigated the moderating effect of gender on relationship of SH and QoL. Previous studies have often investigated the relationship between gender as a contextual and demographic variable with SH or QoL, and have also provided conflicting results. For example, Rajabi et al. showed that there was no significant relationship between gender and SH,^14^ but Keshavarz reported a significant relationship between gender and happiness.^67^ Another study among Iranian paramedical students showed that there was no relationship between happiness and gender.^68^ Rajabi Gilan et al., showed that there was no significant relationship between gender and dimensions of HRQoL.^8^ Lee showed that gender had a significant effect on the elderly's QoL, where elderly men reported a better QoL than women, which is inconsistent with the present study.^69^ Our findings showed that the direct effect of happiness on the perceived QoL was more in men than in women. Considering that in the present study, the score of SH and perceived QoL in women was significantly higher than in men, it seems that part of this finding can be caused by the difference in the biological and emotional characteristics of women compared to men. Another study by Kim et al. indicate that while the quality of social relationships strongly predicts happiness between Korean men and women, however, the path of moderating effect is different according to gender.^70^

Women are more sympathetic and empathetic and give more importance to social interactions and emotional bonds, so their satisfaction with life and feeling happy may depend on their satisfaction with their social, family, and marital relationships.

Our findings showed that gender did not have a moderating effect on the relationship between perceived QoL and PA, although there was a significant relationship between gender and PA, where the score of PA was significantly higher in men. The results of a study in Iran showed that insufficient PA among women and men had a significant difference, which is consistent with the results of the present study.^71^ A study among Turkish teenagers in 2017 showed that there was a significant difference between the PA level of boys and girls.^72^ The results of various studies in Iran have shown that women and girls face many obstacles for sports, which are different according to population and place of residence. Some of these barriers include lack of time, interest and motivation, financial resources, transportation and facilities, social support, and low skill level. Regardless of historical and political issues, traditional values and religion play a much stronger role in the amount and pattern of PA in Iranian women than in men.^73^

This study also showed that gender did not have a moderating effect on the relationship between trust and perceived QoL. There was also no significant relationship between gender and the ST. Rajabi Gilan et al., showed that the relationship between SC and QoL was significant in a sample of teachers after controlling gender variable,^8^ showing this relationship was not affected by gender, which is consistent with the present study to some extent. Another study in Iran showed that there was no significant difference in the average score of SC in terms of family and relatives between men and women, consistent with our study, while there was a significance difference between men and women in terms of the average SC from friends, neighborhood and total SC.^74^

### 4.1 Strength and limitations

The present study used a suitable sample size in a wide geographical area in western Iran and its results are reliable. The study benefited from a determined and coherent theoretical framework and the statistical test used was also appropriate. Studies on the moderating role of gender in relation to the determinants of QoL in Iran are limited. Emphasizing the role of gender, this study has been able to deal with the relationship between social and psychological variables on the QoL. However, this study also had limitations that should be mentioned. In this study, due to its cross‐sectional nature, we only examined the moderating effect of gender and did not investigate the confounding or moderating effect of other variables such as age, education level, and marital status. Although we controlled these items through statistical tests, it is suggested that these variables be examined independently in future studies. A cross‐sectional study can also create limitations due to over‐reporting and under‐reporting of variables. It should be mentioned that this study was conducted in five western provinces of Iran, where people spoke different languages such as Kurdish, Luri, and Persian, and to overcome this limitation, questionnaires were used in Persian as the official language of Iran. Because language barriers and potential translation errors can affect the accuracy and reliability of the data collected, the researchers did not have the possibility to prepare questionnaires in minority languages and this may have affected some of the results. Also, due to cultural and political restrictions and insufficient research budget, the samples were selected by available sampling method and in high‐density places, which may cause bias in the average scores of the main variables. Conducting the study only in urban areas is a limitation of the study, which limits the ability to generalize the results to the entire population.

## 5 CONCLUSION

The results of this study showed that the score of QoL and SH in women was significantly higher than that of men. On the other hand, the mean score of PA in men was higher than that of women. This shows that regarding the SH and PA, there are gender inequalities in the west of Iran.

The results also showed that women's perceived QoL was more than men's. This gender‐based difference needs to be given special attention because the happiness and QoL of men is far less than that of women and can negatively affect men's health. Analyzing the model of the study showed that in the total sample, SH, ST, and social activity had a direct effect on PA. However, gender had an effect on the QoL only through happiness (in favor of men) and did not have a significant effect on ST and PA on QoL. These results indicate that in western Iran, although happiness in men is low, it has a greater role in explaining the QoL.

## AUTHOR CONTRIBUTIONS

**Rajabi Gilan**: Conceptualization; project administration. **Jamal Mohamadi**: Conceptualization; writing—review and editing. **Shirin Zardoshtian**: Conceptualization. **Neda Sarabi**: Formal analysis. **Naseri Palangard**: Formal analysis. **Mehdi Khezeli**: Writing—original draft.

## CONFLICT OF INTEREST STATEMENT

The authors declare no conflict of interest.

## TRANSPARENCY STATEMENT

The lead author Jamal Mohamadi affirms that this manuscript is an honest, accurate, and transparent account of the study being reported; that no important aspects of the study have been omitted; and that any discrepancies from the study as planned (and, if relevant, registered) have been explained.

## Data Availability

The data sets used and analyzed in this study are available from the corresponding author on reasonable request.
